# Invasive lobular carcinoma of the breast: A special histological type compared with invasive ductal carcinoma

**DOI:** 10.1371/journal.pone.0182397

**Published:** 2017-09-01

**Authors:** Zheling Chen, Jiao Yang, Shuting Li, Meng Lv, Yanwei Shen, Biyuan Wang, Pan Li, Min Yi, Xiao’ai Zhao, Lingxiao Zhang, Le Wang, Jin Yang

**Affiliations:** 1 Department of Medical Oncology, The First Affiliated Hospital of Xi’an Jiaotong University, Xi’an, People’s Republic of China; 2 Department of Breast Surgical Oncology, The University of Texas MD Anderson Cancer Center, Houston, Texas, United States of America; University of North Carolina at Chapel Hill School of Medicine, UNITED STATES

## Abstract

The clinical outcomes and therapeutic strategies for infiltrating ductal carcinoma (IDC) and infiltrating lobular carcinoma (ILC) are not uniform. The primary objectives of this study were to identify the differences in the clinical characteristics and prognoses between ILC and IDC, and identify the high-risk population based on the hormone receptor status and metastasis sites. The Surveillance, Epidemiology, and End Results Program database was searched and patients diagnosed with ILC or IDC from 1990 to 2013 were identified. In total,796,335 patients were analyzed, including 85,048 withILC (10.7%) and 711,287 withIDC (89.3%). The ILC group was correlatedwith older age, larger tumor size, later stage, lower grade, metastasis disease(M1) disease, and greater counts ofpositive lymph nodesandestrogen-receptor-positive (ER)/progesterone receptor-positive (PR) positive nodes. The overall survival showed an early advantage for ILC but a worse outcome after 5 years. Regarding the disease-specific survival, the IDC cohort had advantages over the ILC group, both during the early years and long-term. In hormone status and metastasis site subgroup analyses, the ER+/PR+ subgroup had the best survival, while the ER+/PR- subgroup had the worst outcome, especially the ILC cohort. ILC and IDC had different metastasis patterns. The proportion of bone metastasis was higher in the ILC group (91.52%) than that in the IDC (76.04%), and the ILC group was more likely to have multiple metastasis sites. Survival analyses showed patients with ILC had a higher risk of liver metastasis (disease-specific survival[DSS]; P = 0.046), but had a better overall survival than the bone metastasis group (P<0.0001). We concluded that the long-term prognosis for ILC was poorer than that for IDC, and the ER+/PR- subgroup had the worst outcome. Therefore, the metastasis pattern and prognosis must be seriously evaluated, and a combination of endocrine therapy and chemotherapy should be considered.

## Introduction

Breast cancer is a heterogeneous disease with multiple prognoses[1]. Infiltrating ductal carcinoma (IDC) and invasive lobular cancer (ILC), which are classified by their different histological structures and progression histories, are two main histological types of breast cancer [2]. IDC accounts for the majority, and ILC only accounts for approximately one tenth of breast cancer [3]. Clinical studies regarding IDC have shown its dominant role in breast cancer[4–6].The incidence of ILC is increasing[7, 8], and studies have evaluated the different clinical characteristics of this disease[9, 10].

The distinguishing clinical and/or genetic features of ILC and IDC are complex and ambiguous [11–13], and can differ histologically. Therefore, the prognostic outcomes of both ILC and IDC remain controversial. The prognosis of ILC has been reported to be better, worse, or the same as that of IDC [13–15].

The differences in characteristicsof ILCcompared with IDC, arealso reflected inthe diagnoses and treatments. Pathologically, ILC is characterized by monotonous small, round, discohesive cells, gathered into instinct clusters, and invading the adjacent tissues[3, 16].ILC is not easy to diagnosedue to itsambiguousmammography and ultrasonography results[17, 18].

Regarding therapy, are some retrospective study indicated thatILC patients benefited more than IDC patients from endocrine therapy [19]. Recently, a study showed thathormone receptor positive IDC and ILC hadsimilar clinical outcomes, butdifferent clinical treatment strategies, andendocrine therapy was used more often forILC patients[20].

Thusfar, studies comparing ILC withIDC were derived mostly from single centers or were population-based studies[10, 21].the Surveillance, Epidemiology, and End Results (SEER; National Cancer Institute, Bethesda, MD, USA)program reported the incidences, stage-matched outcomes, and the heterogeneous factors of IDC and ILC[13, 22–24]. However, the data was limited tobefore 2009. This study used a population-based database to overcome the limitationsof sample size. Clinical characteristics and survivals were compared between ILC and IDC. Furthermore,we compared the different clinical outcomes of subgroups of ILC and IDC,depending on their hormone receptor status. Finally,endocrine therapystrategies were evaluated, especially for the patients in the high-risk groups.

## Materials and methods

### Patient selection and data collection

**Fig 1:**
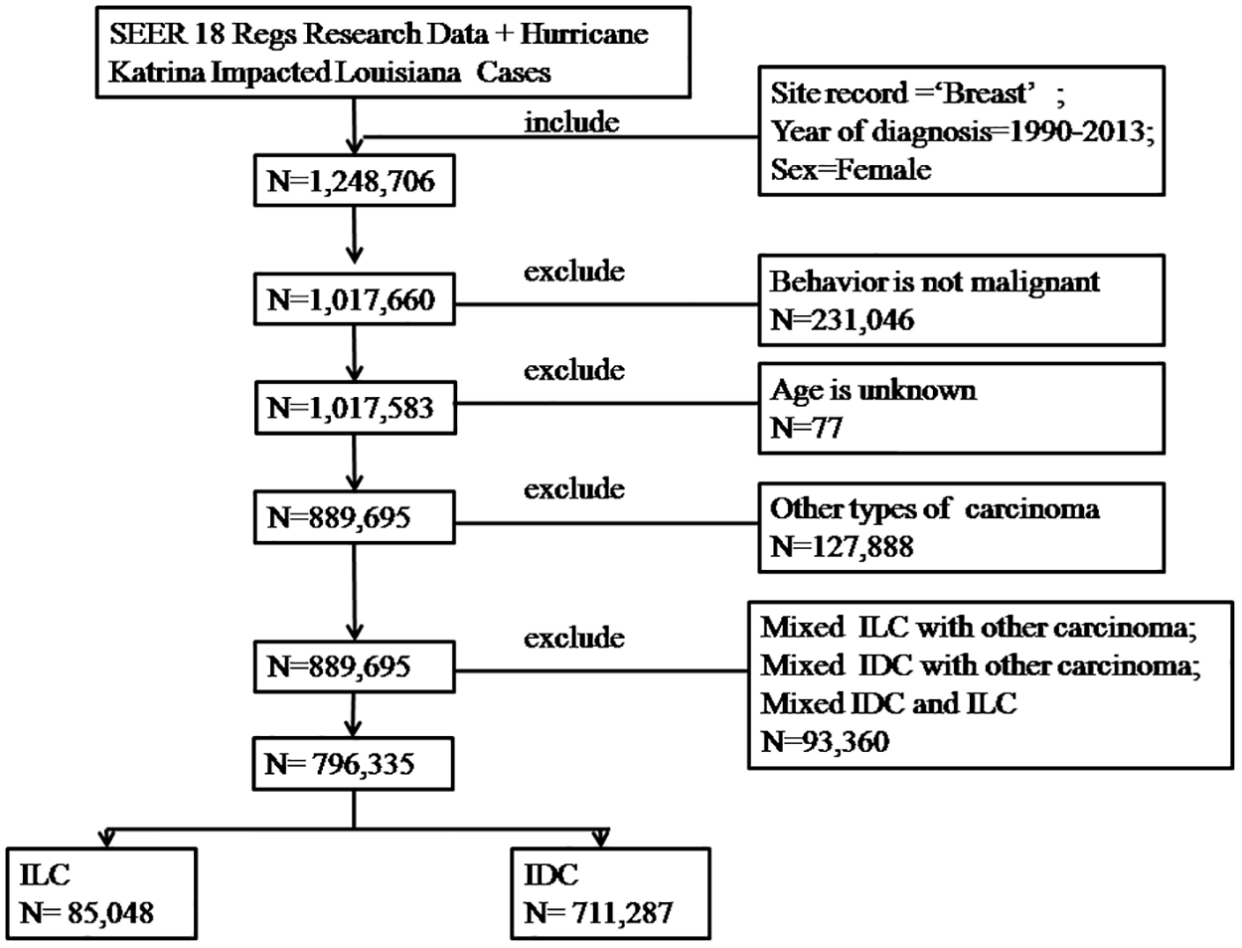
Flow diagram for patient selection. The Surveillance Epidemiology and End Results (SEER) 1990–2013 database was used to identify patients diagnosed with breast carcinoma. Patients were excluded if their disease was not malignant or the age was unknown. And other histology types of disease, including mixed disease were excluded. As the result, the patients were divided into two groups, infiltrating ductal carcinoma (IDC) and infiltrating lobular carcinoma (ILC).

The methods used in this study were similar to those previously reported for the analysis of ethnic differences in lung and bronchial cancer [25]. The SEER database (National Cancer Institute) used the SEER*Stat software program (version 8.3.2; http://seer.cancer.gov/seerstat) with a data user agreement. Patient records were anonymized and de-identified prior to analyses. Because the data were de-identified and from a third party, no ethics committee review approval was required. Patients were identified from the SEER database whose primary tumor sites were coded the ICD-O-3/WHO 2008 as breast cancer diagnosed from 1990 to 2013. We chose histological codes for lobular carcinoma (8520/3) and duct carcinoma (8500/3). Other histological types, like papillary cystadenocarcinoma, squamous cell carcinoma and so on were excluded. Patients with mixed disease (coded as 8522/3) were also excluded (Fig 1). The data for patient age, material status, collaborative stage (CS) tumor size, stage, grade, tumor, node and metastasis (TNM) stage, regional nodes positive status, estrogen-receptor-positive (ER)/progesterone receptor-positive (PR)/human epidermal growth factor receptor 2 (HER2) status, cancer-related surgery status, and radiation follow-up were identified.

### Statistical analyses

We emphasized the comparisons of tumor characteristics and survival of IDC and ILC patients. The patients were primarily categorized into IDC and ILC histological groups. The chi-square test and Kruskal-Wallis test were used to assess differences in clinical characteristics among the groups. The primary endpoint of this study was overall survival (OS) and disease-specific survival (DSS). OS was defined as the number of months from the date of diagnosis to the date of death from any cause.DSS was defined as the number of months from the date of diagnosis to the date of death from breast cancer. OS and DSS curves for the study patients were calculated using the Kaplan-Meier method. Multivariable Cox proportional hazards models were used to determine the influence of relative factors, such as age, tumor size, grade, stage, and positive lymph node hormone status of known or potential prognostic value on survival. All data were analyzed using Stata/SE software (version 12; StataCorp, College Station, TX, USA) and SPSS Statistics software for Windows(SPSS Inc., Chicago, IL, USA). All tests were two-tailed, and statistical significance was set at P<0.05.

## Results

### Comparisons of clinical characteristics by histological group

In total,796,335 patients diagnosed with ILC or IDC were analyzed in the study. Among the study population, 85,048 had ILC (10.7%) and 711,287 had IDC(89.3%).

The clinical characteristics ofthetwo histologicalsubtypes are summarized in Table 1.Significant differences in constituent ratio of the age,marital status, tumor site counts, size, stage, grade, TNM stage, hormorne receptors status, lymph node counts, surgery and radiation therapy were observed. ILC patients were olderat diagnosisthan IDC patients (>60 years of age, 63.4% and 52.3% respectively). The ILC grouppatients tended to had tumorthat were larger, later stage, and lower gradethan thosein the IDC group. The percentageof metastasis disease(M1) when diagnosed was higher in the ILC group(ILC vs IDC, 5.5% vs 3.8%). The counts of positive lymph nodeswere also higher in the ILC group (P<0.0001). Patients inthe ILC group were more likely to be ER positive and PR positive. In addition, the selection of surgical methods differed between the groups; the ILC group had ahigher percentage of mastectomiesthan the IDC group (50.7% vs 40.3%;P<0.0001), while the IDC group was dominated by breast-conserving surgery.

**Table 1:** Baseline demographic and clinicopathologic characteristics of the 796,335 study patients.

Characteristic	ILC% (n = 85,048)	IDC % (n = 711,287)	P
**Age at diagnosis, years**			<0.0001
<40	1.7	5.9	
40–60	34.9	41.8	
>60	63.4	52.3	
**Marital status**			0.001^c^
Single	45.0	42.9	
Married	55.0	57.1	
**Tumor site(s)**			<0.0001 ^c^
Only one site (left or right)	99.6	99.9	
Two or more sites	0.4	0.1	
**Tumor size, mm**			<0.0001 ^c^
No tumor	0.3	0.1	
< = 20	50.7	61.5	
21–40	27.9	26.8	
> = 41	21.1	11.7	
**Tumor stage**			<0.0001 ^c^
0	0.0	0.0	
I-II	76.8	83.7	
III-IV	23.2	16.3	
**Tumor grade**			<0.0001 ^c^
I	28.4	18.2	
II	57.0	41.6	
III	13.7	38.7	
Undifferentiated	0.9	1.5	
**Breast–Adjusted AJCC 6**^**th**^ **T**			<0.0001 ^c^
T0	0.0	0.0	
Tis	0.0	0.0	
T1-2	81.6	89.5	
T3-4	12.4	6.4	
Any T, Mets	5.9	4.1	
**Breast–Adjusted AJCC 6**^**th**^ **N**			<0.0001
N0	59.1	62.0	
N1	18.9	21.0	
N2	7.0	6.1	
N3	6.4	3.8	
NX	8.6	7.1	
**Breast–Adjusted AJCC 6**^**th**^ **M**			<0.0001
M0	92.3	94.2	
M1	5.5	3.8	
MX	2.2	1.9	
**Lymph node, positive counts**			<0.0001 ^c^
Negative	63.2	66.6	
1–3	21.1	22.8	
4–10	9.6	7.8	
>10	6.0	2.8	
**ER status**			<0.0001 ^c^
positive	94.8	76.6	
negative	5.0	23.1	
Borderline	0.2	0.3	
**PR status**			<0.0001 ^c^
positive	78.1	65.7	
negative	21.2	33.6	
Borderline	0.7	0.6	
**ER/PR status**^b^			<0.0001
ER+/PR+	77.8	64.5	
ER+/PR-	17.2	12.3	
ER-/PR+	0.9	1.8	
ER-/PR-	4.1	21.5	
**HER2 status**^a^			<0.0001
positive	4.8	16.4	
negative	93.5	81.1	
Borderline	1.7	2.4	
**Cancer directed surgery**			<0.0001 ^c^
Not performed	8.3	6.3	
Breast conserving surgery	40.9	53.2	
Mastectomy	50.7	40.3	
Surgery, NOS	0.2	0.1	
**Radiation**			<0.0001 ^c^
Not performed	57.6	52.5	
Performed	42.4	47.5	

ILC, invasive lobular carcinoma; IDC, invasive ductal carcinoma; ER, estrogen receptor; PR, progesterone receptor; -, negative; +, positive; NOS, not otherwise specified.

^a^Statistic data only included the patients who had HER2 records after 2010.

^b^Calculated after exclusion of patients with the unknown or borderline ER/PR status.

^c^ Calculated after exclusion of patients with the unknown groups.

### OS and DSS of ILC and IDC

**Fig 2:**
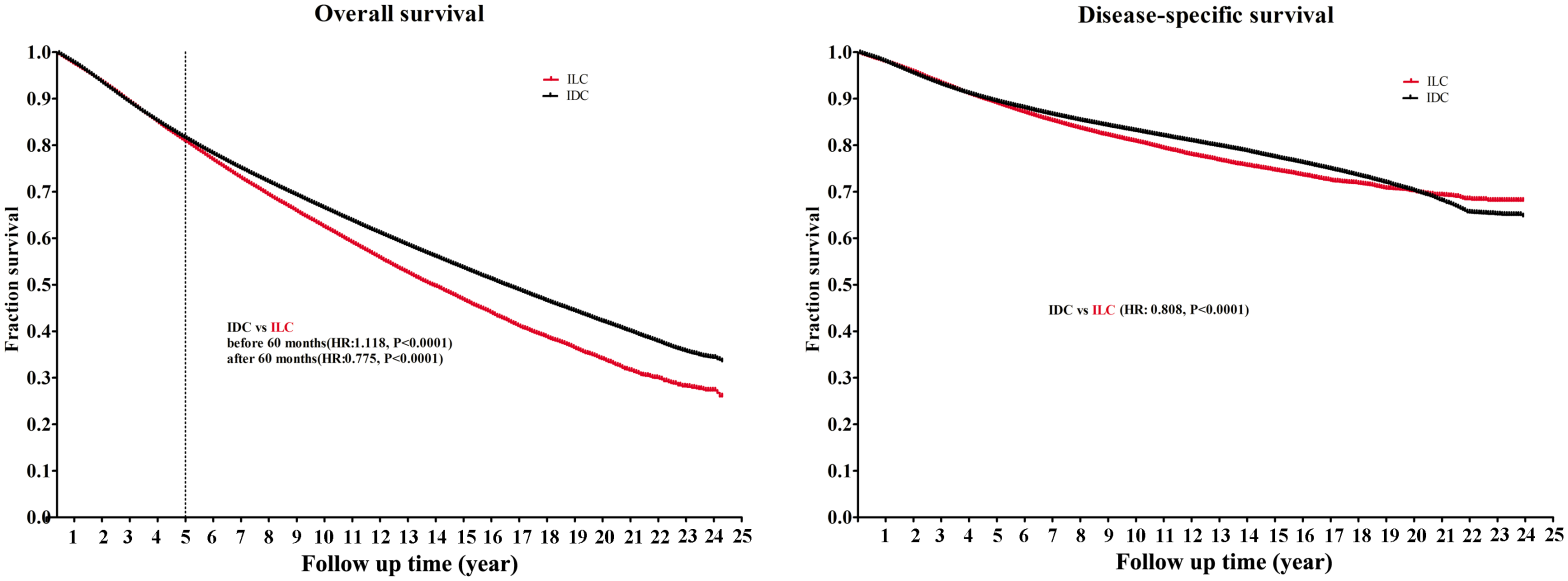
Comparison of overall survival (OS) rates and disease-specific survival (DSS) rates of infiltrating ductal carcinoma (IDC) and infiltrating lobular carcinoma (ILC).

In this study, the median follow-up duration was 5.5 years (mean, 6.8years; range, 0 to 24years). Fig 2 shows the OS and DSS of ILC and IDC. The OS curve showed an early advantage for the ILC patients before 60 months (ILC vs. IDC, hazard ratio [HR], 1.118; P<0.0001). However, after 5 years, an advantage for the IDC group was observed (ILC vs. IDC, HR, 0.775; P<0.0001). Regarding the DSS curve, the IDC group had a better survival than the ILC group, both early and long-term (ILC vs. IDC, HR, 0.809; P<0.0001).

Cox proportional models were used to assess the clinicopathological factors related to survival (Table 2).Tumor size(>20mm), tumor stage(III–IV), tumor grade(II–III), positive lymph node counts, distant metastasis(M1) when diagnosed, and hormone receptors negative/borderline status were poor prognostic factors for ILC. Apart from the factors identified in the ILC group, patients in the IDC group had additional adverse prognostic factors including older age (>40-years-old), undifferentiated tumor grade, and T stage. Surgery and radiation were two protective factors for survival. Among the patients receiving surgery, breast-conserving surgery was more favorable for survival than mastectomy (ILC, HR:0.69; IDC, HR:0.63, P<0.0001)

**Table 2:** Multivariable cox proportional hazards analysis of clinicopathologic factors associated with survival, stratified by histology types.

	ILC (n = 85,048)				IDC (n = 711,287)			
Factor	HR	P	95% CI		HR	P	95% CI	
Age at diagnosis, years								
<40	Referent				Referent			
40–60		NS	0.64	1.42	1.20	<0.0001	1.09	1.31
>60		NS	1.00	2.19	2.92	<0.0001	2.67	3.19
Marital status								
Single	Referent				Referent			
Married	0.71	<0.0001^a^	0.64	0.80	0.63	<0.0001^a^	0.61	0.65
Tumor site(s)								
Only one site(left or right)	Referent				Referent			
Two or more sites		NS	0.46	1.20		NS	0.57	1.04
Tumor size, mm								
< = 20	Referent				Referent			
21–40	1.37	<0.0001^c^	1.19	1.59	1.45	<0.0001^c^	1.39	1.51
> = 41	1.28	<0.0001^c^	1.09	1.51	1.49	<0.0001^c^	1.41	1.57
Tumor stage								
I-II	Referent				Referent			
III-IV	1.79	<0.0001^c^	1.41	2.29	1.16	0.001^c^	1.06	1.26
Tumor grade								
I	Referent				Referent			
II	1.34	<0.0001^a^	1.17	1.54	1.16	<0.0001^a^	1.10	1.21
III	1.83	<0.0001^a^	1.53	2.17	1.58	<0.0001^a^	1.51	1.67
Undifferentiated		NS^a^	0.75	4.45	1.75	<0.0001^a^	1.45	2.11
Breast–AdjustedAJCC 6^th^ T								
T0	Referent				Referent			
Tis		NS^a^	0.08	1.68		NS^a^	0.54	2.76
T1-2		NS^a^	0.01	2.18	3.43	0.005^a^	1.45	8.13
T3-4		NS^a^	0.34	7.07	4.17	0.001^a^	1.85	9.44
Any T,Mets		NS^a^	0.15	3.81		NS^a^	0.71	4.16
Breast–AdjustedAJCC 6^th^ N								
N0	Referent				Referent			
N1		NS^a^	0.77	1.14	0.87	<0.0001	0.81	0.93
N2		NS^a^	0.69	1.26	0.89	0.023	0.80	0.98
N3		NS^a^	0.80	1.22		NS^a^	0.95	1.12
NX		NS^a^	0.73	1.16	1.12	0.032	1.01	1.24
Lymph node, positive counts								
Negative	Referent				Referent			
1–3	2.50	<0.0001^a^	1.19	3.27	1.62	<0.0001^a^	1.50	1.76
4–10	3.95	<0.0001^a^	2.78	5.61	2.38	<0.0001^a^	2.15	2.65
>10	5.98	<0.0001^a^	4.35	7.98	3.11	<0.0001^a^	2.78	3.46
Positive, counts unknown	4.68	<0.0001^a^	3.39	6.48	2.10	<0.0001^a^	1.89	2.34
No examined nodes	6.95	<0.0001^a^	3.46	13.9	3.12	<0.0001^a^	2.50	3.91
Breast–AdjustedAJCC 6^th^ M								
M0	Referent				Referent			
M1	3.11	<0.0001	2.62	3.71	2.68	<0.0001	2.55	2.83
MX		NS	0.93	1.97		NS	0.93	1.14
ER status								
Positive	Referent				Referent			
Negative	2.14	<0.0001^a^	1.82	2.52	1.87	<0.0001^a^	1.82	1.92
Borderline	5.24	<0.0001^a^	1.95	14.1	1.93	<0.0001^a^	1.48	2.52
PR status								
Positive	Referent				Referent			
Negative	2.08	<0.0001^a^	1.89	2.29	1.70	<0.0001^a^	1.65	1.74
Borderline	2.51	<0.0001^a^	1.51	4.20	1.28	0.007^a^	1.07	54
ER/PR status								
ER+/PR+	Referent				Referent			
ER+/PR-	1.98	<0.0001^b^	1.79	2.20	1.37	<0.0001^b^	1.32	1.43
ER-/PR+	2.55	<0.0001^b^	1.52	4.29	2.02	<0.0001^b^	1.47	2.23
ER-/PR-	1.95	<0.0001^b^	1.81	2.11	1.79	<0.0001^b^	1.76	1.83
Cancer directed surgery								
Not performed	Referent				Referent			
Performed	0.16	<0.0001^a^	0.15	0.17	0.17	<0.0001^a^	0.17	0.17
Surgery category								
Mastectomy	Referent				Referent			
Breast conserving surgery	0.69	<0.0001^a^	0.66	0.71	0.63	<0.0001^a^	0.62	0.64
Radiation								
Not performed	Referent				Referent			
Performed	0.73	<0.0001^a^	0.65	0.82	0.58	<0.0001^a^	0.56	0.60

HR, hazard ratio; CI, confidence interval; NS, not significant; ILC, invasive lobular carcinoma; IDC, invasiveductal carcinoma;ER, estrogen receptor; PR, progesterone receptor; -,negative; +, positive; NS, not significant.

^**a**^ Calculated after exclusion of patients in the Unknown category;

^**b**^ Calculated after exclusion of patients in the Borderline or Unknown category.

^**c**^Calculated after exclusion of patients in the no tumor, stage 0 or Unknown category.

There were some additional findings of note. First, old age and T stage were not unfavorable factors for the survival of ILC patients. Second, metastasis factors, including M stage or lymph node counts, had more pronounced impacts on the prognosis of ILC. For example, the HRs of lymph node positive (>10) vs. lymph node negative were 5.98 and 3.11 in the ILC and IDC groups, respectively. In addition, hormone receptor status played role in the survival of the patients in the two groups, but the ILC cohort was influenced to a greater extent.

### Comparisons of survival in the ILC and IDC patients subgrouped by ER/PR status

**Fig 3:**
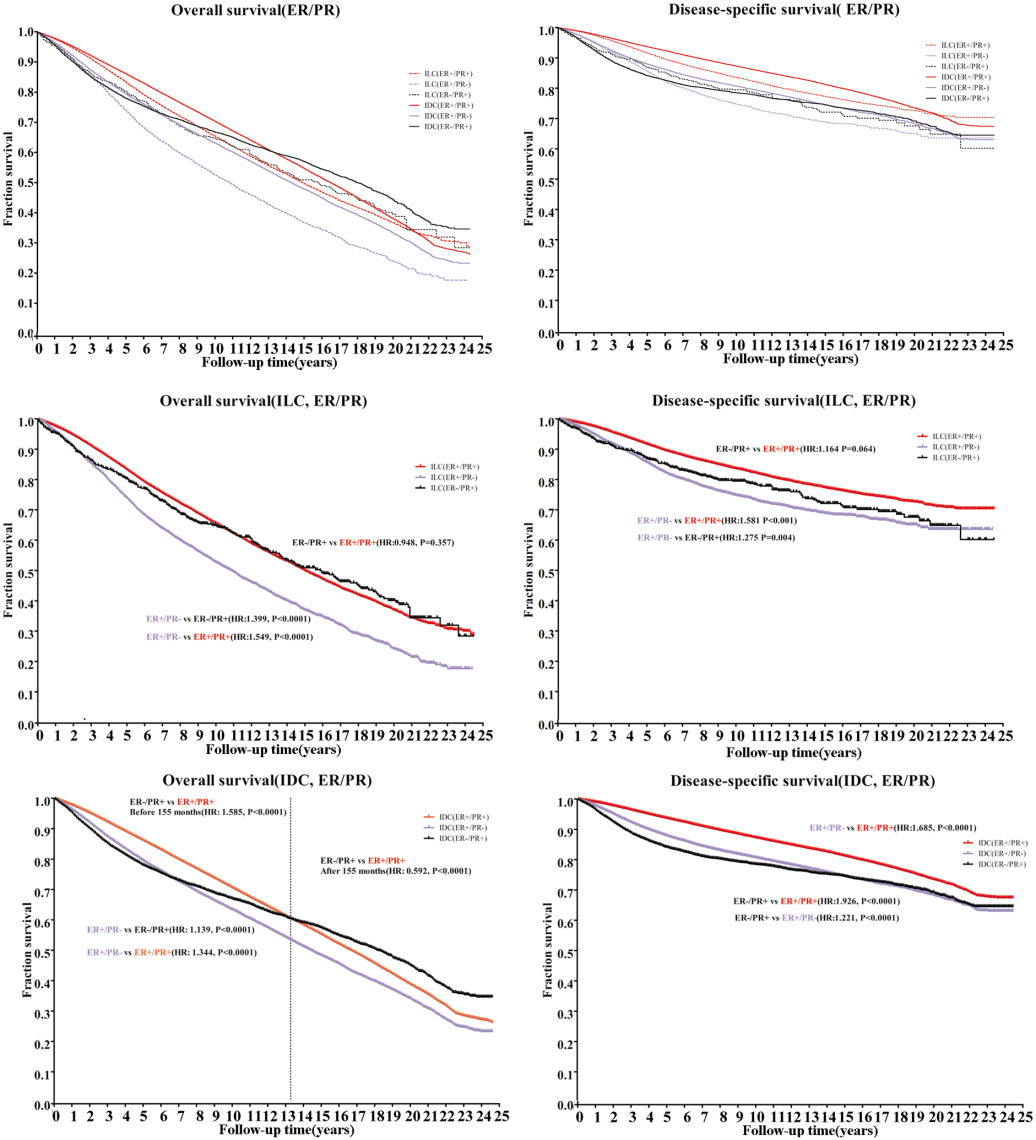
Comparison of overall survival (OS) rates and disease-specific survival (DSS) ratesof infiltrating ductal carcinoma (IDC) and infiltrating lobular carcinoma (ILC) based on hormone receptor status.

Based on the results that the ILC cohort was dominated by hormone receptor positive patients, and hormone receptors are important factors for prognosis as well as clinical strategy making, patients diagnosed with ER-/PR- were excluded because of the relatively small population and the entirely different clinical management. We performed analyses of the following subpopulations: ER+/PR+, ER+/PR-, and ER-/PR+. Fig 3 shows the different OS and DSS curves of the subgroups in the ILC and IDC cohorts. In the ER+/PR+ and ER+/PR- subgroups, the prognosis of IDC were better than that for ILC, regardless of OS or DSS(Table 3).

**Table 3:** OS and DSS comparison in different hormone receptor status of ILC and IDC.

	ILC^a^ vs IDC							
Hormone receptor status	OS				DSS			
	HR	P	95%CI		HR	P	95%CI	
ER+/PR+	0.92	<0.0001	0.91	0.94	0.66	<0.0001	0.64	0.67
ER+/PR-	0.81	<0.0001	0.79	0.84	0.71	<0.0001	0.68	0.74
ER-/PR+		NS	0.82	1.03		NS	0.90	1.25

^a^The ILC was chose to be the referent and the prognosis were compared within every subgroup.

HR, hazard ratio; CI, confidence interval; NS, not significant; ILC, invasive lobular carcinoma; IDC, invasive ductal carcinoma;ER, estrogen receptor; PR, progesterone receptor; -,negative; +, positive; NS, not significant.

In order to measure the different influence of hormone status on the histological subgroups, we first analyzed the curves separately by histological types (Fig 3). For the OS of the ILC cohort, the risk of death in the patients with ER+/PR- (purple dotted line) tumors was 50% higher than those with ER+/PR+(red dotted line) tumors(HR: 1.549;P<0.0001), and was 40% higher than those with the ER-/PR+ (black dotted line) tumors(HR:1.399;P<0.0001). A similar pattern was found in the DSS curves of the ILC group. The IDC group showed a different survival pattern based on ER/PR status. The ER+/PR+ subgroup(red solid line) showed a survival advantage in DSS over the ER-/PR+ subgroup (black solid line)(ER-/PR+ vs. ER+/PR+,HR: 1.926;P<0.0001) and ER+/PR-(purple solid line)(ER+/PR- vs. ER+/PR+,HR: 1.685;P<0.0001), which seemed to be more pronounced than that in the ILC group. Crossed patterns of ER-/PR+ with the other two subgroups were seen for OS in the IDC cohort. Before 13 years, the survival of the ER-/PR+ subgroup patients was worse than that of the ER+/PR+ subgroup (HR: 1.585;P<0.0001), andthe trend was worse with increasing follow up time (HR:0.592;P<0.0001). The trendwas not observed in DSS analyses.

In summary, the ER+/PR+ subgroup (red line) had the best survival, while the ER+/PR- subgroup (purple line) had the worst survival, whether determined as OS or DSS. The ER+PR- subgroup in ILC cohort had the worst survival with respect to OS and DSS(P<0.0001).The ER-/PR+ subgroups in the ILC and IDC cohorts had similar survivals as the ER+/PR- subgroup in the IDC cohort regarding DSS, and seemed to have an advantage in survival over that demonstrated in the OS curve. These results meant that when ER and PR were expressed together at a different level, the clinical outcome differed between the subgroups in ILC and IDC, and the ER+/PR- subgroup in the ILC had the worst prognosis.

**Fig 4:**
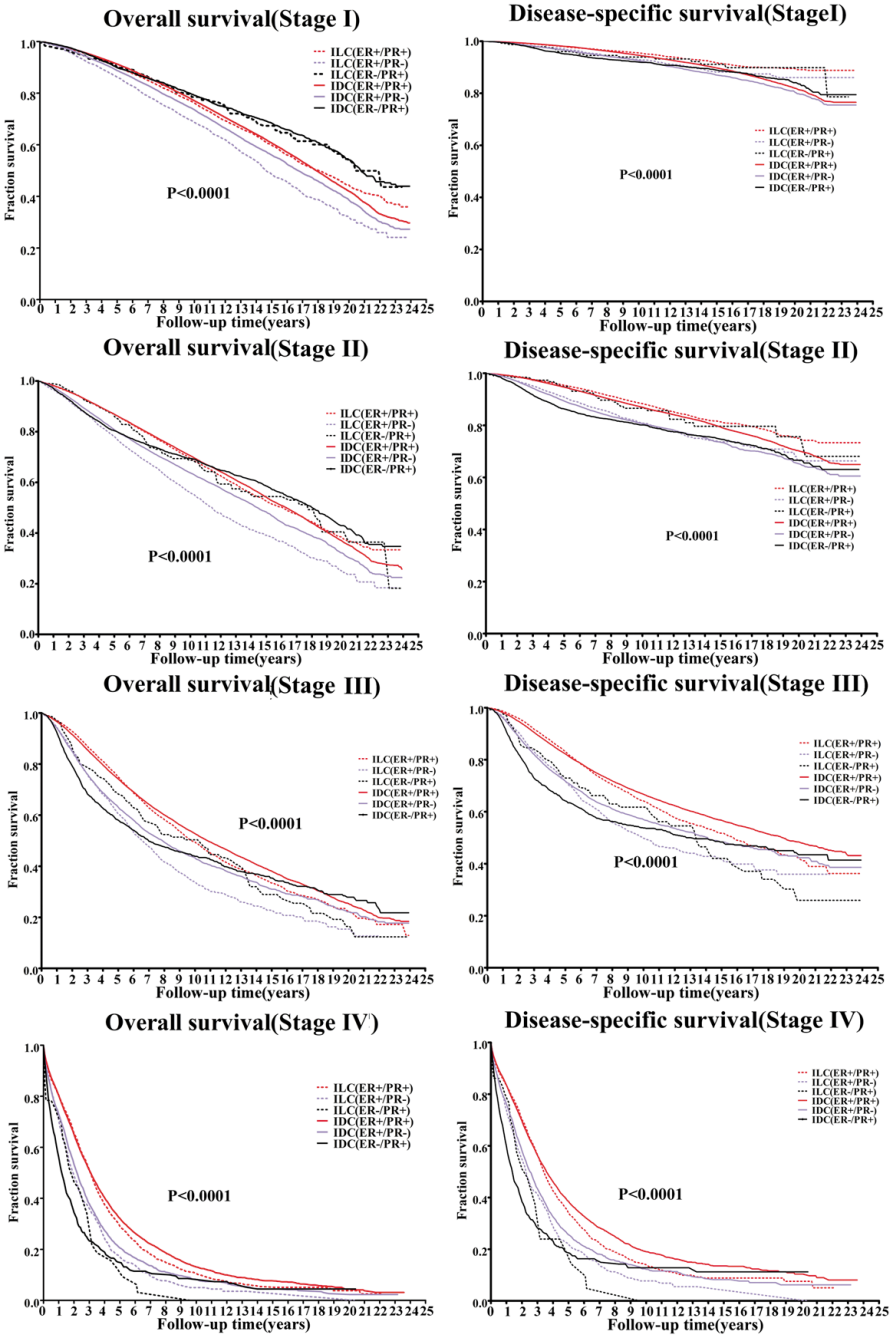
Kaplan–Meier survival curves of infiltrating ductal carcinoma (IDC) and infiltrating lobular carcinoma (ILC) based on hormone receptor statusmatched by stage. OS, overall survival; DSS, Disease-specific survival.

In order to compare the patients’ groups, we further matched the subgroups by tumor stage (Fig 4). The disparity of the clinical outcomes was greater with staging. At an early stage, there was a slight advantage in the DSS of the ILC group. However, the OS of the ILC patients was poorer than that of the IDC patients. At a late stage, the prognosis of ILC was worse than IDC. Taking stage and receptor status together, the outcome of the ER+/PR- subgroup changed with the tumor stage, which appeared worse in stages I–II, but better in stage IV. The ER-/PR+ subgroup showed a better outcome in OS and DSS at an early stage, but had the worst prognosis at stage IV.

### The metastasis sites and counts

The SEER database recorded the metastasis data of patients diagnosed with M1 disease after 2010. Therefore, we calculated the proportion of metastasis sites (bone, brain, liver, and lung) and the site counts in ILC and IDC separately. Table 4 showsthe statistical results. In total, 8,639 patients (1,128 ILC and 7,511 IDC) had metastatic disease in their primary visit after 2010, with records of metastasis sites. Bone metastasis was the most common in ILC and IDC, but the proportion was higher in the ILC group (91.52%) than that in the IDC group (76.04%). The second most common metastasis site was the liver (19.64%), followed by the lung (13.61%) and brain (4.23%) in the ILC group while the order in the IDC group was the lung (37.11%), liver (30.53%) and brain (8.24%).

**Table 4:** Comparison of infiltrating ductal and infiltratinglobular histological types according to the metastasis sites and counts.

	ILC N = 1,128^a^		IDC N = 7,511^a^		P
	No. of Patients	%	No. of Patients	%	
**metastasis sites**					<0.0001
Bone	1,123	91.52	5444	76.04	
Brain	521	4.23	619	8.24	
Liver	241	19.64	2293	30.53	
Lung	167	13.61	2787	37.11	
**Counts of metastasis sites**					<0.0001
One	933	76.04	4706	62.65	
Two or more	195	23.96	2805	37.35	

ILC, invasive lobular carcinoma; IDC, invasive ductal carcinoma;

^a^The statistic data only include the patients who had metastasis site records when diagnosed.

In addition to the metastasis pattern differences between these two histological groups, the counts of metastasis sites when diagnosed were also different. The percentage of patients with only one metastasis site was higher in the ILC group. The IDC group usually had more than one metastasis site (37.35% vs. 23.96%; P<0.0001).

### Comparison of survival in the ILC and IDC subgrouped by metastasis sites

**Fig 5:**
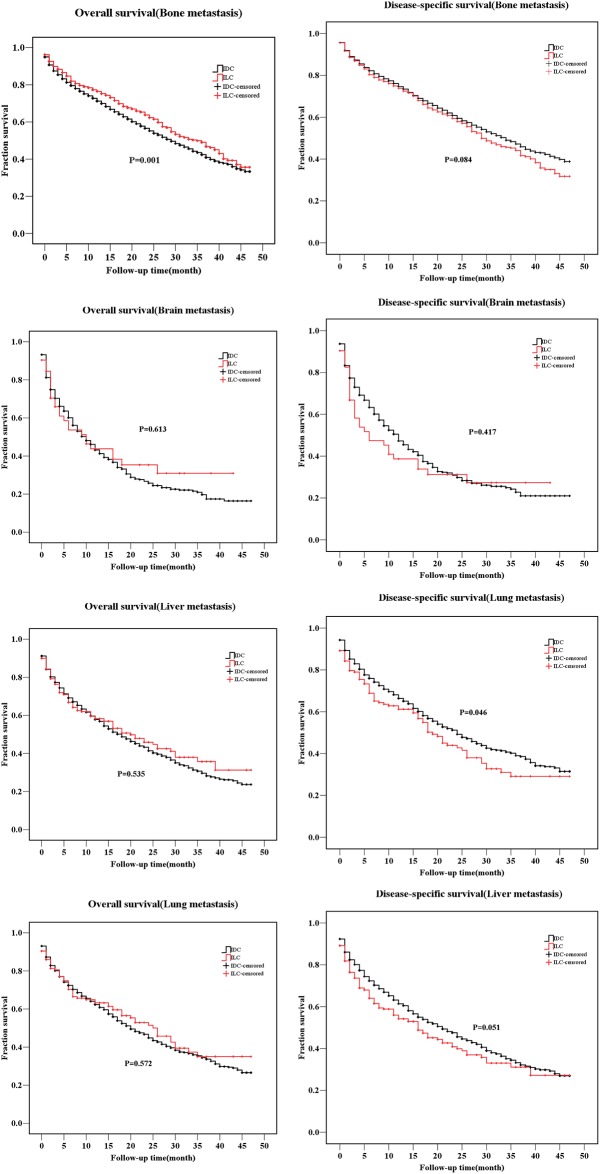
Comparison of overall survival (OS) rates and disease-specific survival (DSS) rates of infiltrating ductal carcinoma (IDC) and infiltrating lobular carcinoma (ILC) stratified by different metasasis sites.

Apart from the hormone receptor status, the metastasis sites also differed, which could be critical for clinicians’ management of the patients. The data for patients who had the metastasis site records in the SEER database were limited from 2010 to 2013, therefore, we analyzed the available data after 2010. Metastatic disease when diagnosed as M1 was an unfavorable factor for survival (Table 2; ILC, HR = 3.11; IDC, HR = 2.68). Taking the histology type into account, we compared the survival of patients with different metastatic disease in the ILC and IDC groups (Fig 5). The ILC group with bone metastasis had a better OS (P = 0.001) than the IDC group, but no significance was found in DSS. Liver metastasis, however, showed a higher risk for ILC patients’ DSS (P = 0.046). A statistical significance was not found in the survival curves for other metastasis sites.

## Discussion

Among the newly diagnosed breast cancer patients, IDC is the majority subtype and ILC only accounts for 5–15% of the patients[8, 26]. However, the incidence of ILC has significantly increased [27]. Compared with IDC, the biological and clinical characteristics of ILC should be taken more seriously in clinical practice. No comparison of long-term survival of the two histological types has been reported. In addition, differences in endocrine therapy duration still exist in systemic treatment protocols. Our study was a comprehensive population-based analysis of the clinical characteristics and outcomes of ILC and IDC. The most recent data were collected to provide relatively objective results. We also provided data regarding targeted systemic therapy, especially endocrine therapy and comprehensive treatments.

We showed that ILC was associated with larger tumor size, older age, later stage, lower grade, ER/PR positive, and lower expressionofHER2. These results were similar to those of studies [28–31]. Higher percentages of lymph node positive and distant metastasis were found in ILC, which has also been previously reported[10]. A delay in diagnosis and failure of detection could be reasons for the more advanced stages of patients compared to the IDC patients [10, 32].In our study population, the patients with ILC received more breast surgery. The same data were reported previously [9], but recent evidence has indicated that breast-conserving surgery is not associated with increased relapse when compared with mastectomy[21].

To the best of our knowledge, the high percentages of mastectomy in ILC were due to the difficulty in the identification of the surgery margin. However, the greater chance of multicenter tumor formation could be another reason.

Our study suggested that the metastatic pattern for ILC differs from that of IDC. A previous study showed that ILC was less likely to affect the lungs, pleura, and central nervous system than IDC [32]. In this study, bone metastases were more common in ILC than IDC patients, and IDC patients had more metastatic disease sites, such as the brain, liver and lung than the ILC patients. In addition, the IDC group had more patients with more than one metastasis sites. The mechanism for this is unclear. However, a loss of expression of the cell–cell adhesion molecule E-cadherin was reported to decrease adhesiveness of cells in ILC [33–35],and the microenvironment of the different organs might facilitate specific tumor cells with the appropriate size and shape to adhere and grow. This could explain the complex pattern of tumor metastasis; however, more in-depth studies are needed.

The current study provided no consensus regarding the survival prognosis of ILC and IDC patients. Classic ILC was reported to have a better prognosis than IDC [36]. A retrospective analysis by Azimet al. showed that ILC was associated with better disease-free survival[37].Recently, another study compared the clinical outcomes between luminal IDC and luminal ILC, and found that luminal ILC had a worse outcome[38].Our study found that ILC had a worse prognosis in long-term survival, both for overall survival and in disease-specific survival, and the related risk factors differed between the subgroups. Multivariate analyses showed that some factors like tumor size, tumor stage, tumor grade, surgery, and radiation therapy similarly affected affect the prognosis of ILC and IDC; but the positive lymph node counts, ER/PR status, and distant metastasis were more important factors for ILC. Although surgery and radiation therapy were two equally favorable factors for ILC and IDC, the percentage of patients receiving the therapy was less in the ILC group, and the proportion of mastectomy in the ILC group was higher. The National Surgical Adjuvant Breast Project (NSABP) has reported equivalent results for breast conserving surgery vs. mastectomy in patients with IDC [39],and some studies of ILC have also been reported[21, 40]. We suggest that breast conserving surgery hasa survival advantage over mastectomy. T stage was also an important factor for the survival of IDC, but not for ILC patients. A study suggested that ILC had a better response to endocrine therapy and a higher risk of metastatic disease [19].

The treatments for patients with ILC are similar to those for patients with IDC, and the prognoses have been reported [32, 41].ILC is often diagnosed as a later stage and larger tumor size, with numerous lymph node positive results. Neoadjuvant chemotherapy was reported as being less effective for ILC patients [10, 42], while aromatase inhibitors were reported to have more benefits to patients with ILC than IDC [43]. In general, endocrine therapy was one of the most important treatments for patients with positive hormone status. Tamoxifen, ovarian function suppression, and anastrozol were used and reported to be effective strategies for patients with luminal type breast cancer[44–46].The aromatase inhibitor was recommended for postmenopausal ILC patients[47], but there still exist some questions concerning the type and the duration of treatment [48]. A definition of what constitutes a patient in the high-risk group still remains to be determined.

This study emphasized the different prognoses of ILC and IDC when grouped by ER/PR status. Our results showed that the ILC subgroup had a higher risk than the IDC group in terms of long-term survival. The ILC patients with hormone receptor status ER+/PR-had a poorer survival than the other groups. ILC patients in late stage had obviously poorer outcome than IDC, regardless of the receptor status. In our study, ILC was associated with a more advanced tumor stage. Tumor stage, hormone receptor status and histological type should be taken into account when evaluating the clinical outcome and therapy strategies of patients. Given that the ER+/PR- subgroup in ILC was the group less sensitive to endocrine therapy, the use of a stronger endocrine therapy procedure, and other comprehensive therapies may need to be considered. Although the follow-up time in patients with metastatic disease was limited, wefound that bone metastatic disease at first was a greater risk to IDC patients in short-term survival, while liver metastasis indicated a poorer prognosis for ILC. Use of chemotherapy might be important for ILC patients having a worse prognosis due to liver metastasis. Bone therapy could also be useful for the high incidence and worse survival of IDC group. Because ILC is more likely to be diagnosed with distant metastasis in the late stage, combination of chemotherapy and hormone therapy may be beneficial. The American Society of Clinical Oncology Guidelines, recommended endocrine therapy as an initial treatment for hormone receptor positive metastatic breast cancers [49].

In summary, this study was based on a population-based cancer registry to overcome a limited sample size. A comprehensive comparison of the clinical characteristics and survival prognoses of ILC and IDC were performed. In addition, in-depth analyses of hormone receptor status and metastasis sites were performed. However, some limitations to the study exist. This was a retrospective study. The data was updated to 2013, but some information regardingHER2and metastasis site records were only available after 2010. Lastly, the recurrence and the treatment information were unavailable.

## Conclusions

ILC should be treated as an independent disease, which has different biological behaviors. The long-term prognosis of ILC is poorer than that of IDC, and the ER+/PR- subgroup has the worst outcome. Metastasis diseases show poor survival, and different histological types differed in their survival depending on the metastasis sites. Endocrine therapy should be increased, and other comprehensive therapies should be considered, especially for the ER+/PR- subgroup of ILC patients. The metastasis pattern and prognosis should be considered when determining the use of combinations of endocrine therapy and chemotherapy.
